# Editorial: Structural Plasticity of Invertebrate Neural Systems

**DOI:** 10.3389/fphys.2022.874999

**Published:** 2022-03-16

**Authors:** Hadley Wilson Horch, Wolfgang Rössler, Gaia Tavosanis

**Affiliations:** ^1^Biology and Neuroscience, Bowdoin College, Brunswick, ME, United States; ^2^Behavioral Physiology and Sociobiology (Zoology II), Biozentrum, University of Würzburg, Würzburg, Germany; ^3^German Center for Neurodegenerative Diseases, Helmholtz Association of German Research Centres (HZ), Bonn, Germany

**Keywords:** plasticity, morphology, adult, neurotrophins (NTs), regeneration, neuroarchitecture

The flexible plasticity of developing nervous systems has been well-documented in a variety of animal species. Adult nervous systems maintain the ability to make structural rearrangements under certain conditions. Generally, invertebrate nervous systems are thought to be less flexible than vertebrate nervous systems, but some surprising examples of extensive adult structural plasticity have been described in invertebrates (Tavosanis, 2012; Sugie et al., 2018; Groh and Rössler, 2020; Anton and Rössler, 2021). Structural changes in response to injury, due to circadian rhythms, or accompanying adaptation or learning have been described in these simpler nervous systems, suggesting that the capacity for flexibility and plasticity might be a key, and thus conserved, trait of evolutionarily distant organisms.

In the present Research Topic in Frontiers on *Structural Plasticity of Invertebrate Neural Systems*, we present seven original papers and reviews illustrative of current directions within this exciting field of research. Some of these papers contribute novel data, laying groundwork for future studies exploring adult plasticity. Others characterize compelling examples of invertebrate adult plasticity in insects. Others explore the role of invertebrate neurotrophins in adult structural plasticity, highlighting an exciting avenue for invertebrate research. And finally an opinion piece proposes an analytical approach to clarify how brain size may have evolved in response to environmental, social, and cognitive requirements. All together, these contributions highlight the capacity for and consequences of neuronal structural plasticity in invertebrates.

Exploration of adaptations and plasticity in mollusk species indicates that homologous genes organize molluscan nervous systems in many different ways, depending on the demands of the environment. As a first step in decoding this variety, Kotsyuba et al. have characterized the distribution of a number of neuropeptides, neurotransmitters, and related enzymes. They find that bivalve nervous systems contain a broad variety of neurotransmitter-related molecules and are next prepared to explore the functional regulation of physiological and behavioral processes. In addition, they found a population of proliferating cells in the nervous system, which they hypothesize maintain and renew glial cells, and possibly neuronal populations, over the lifespan of these organisms.

Bicker and Stern review compelling examples of regeneration and plasticity in the olfactory system of the locust. They discuss experiments in which axotomized olfactory afferents induce the subsequent degeneration and regeneration of the antennal lobe, including data showing the volume of this lobe was reduced after deafferentation but recovered when the afferent axons regenerated. In this system, the return of odor responses after regeneration confirm the re-establishment of synapses. This model of precise and rapid axonal regeneration can now be explored mechanistically to understand how nervous systems are capable of such specific neuronal recovery.

Studies on the neural control of locomotion have been key to learning more about the plasticity of invertebrate nervous systems. Aponte-Santiago and Littleton review the general properties of invertebrate tonic and phasic motorneurons, highlighting the historic use of crustacean models along with the modern genetic approaches possible in Drosophila today. This insect system offers a chance to explore how distinct transcriptional programs generate the different firing properties, unique synaptic structures, and the plasticity mechanisms that distinguish these two neuronal subgroups.

Exploring the concept of sensory plasticity and locomotion, Miguel-Blanco and Manoonpong took a robotics approach. They developed a learning mechanism in walking robots to introduce plasticity between the sensory feedback and the neural circuitry controlling locomotion that was continuous, online, and fast. This resulted in the generation of stable self-organized locomotion, which could be adapted to different types of walking robots. Robots with this plasticity mechanism were also able to deal with damage within a few walking steps, which Miguel-Blanco and Manoonpong categorize as being informative for lesion-induced plasticity. This response can be instructive for examples of injury-induced plasticity seen in invertebrates.

The molecular mechanisms responsible for structural plasticity in the adult invertebrate brain are only just coming into focus. The discovery of several different types of neurotrophin-like proteins in invertebrates provides potential mechanisms for regulating cell number and neuronal morphology in the adult brain. Li and Hidalgo highlight the growing evidence that invertebrate neurotrophin ligands bind Toll receptors to regulate structural plasticity both during development and in the adult brain. They review the Toll signaling pathway and its role in brain size, structural homeostasis, circadian plasticity, and control of cell number in *Drosophila*. Krzeptowski et al. explore the role of a different invertebrate neurotrophic factor, Mesencephalic Astrocyte-Derived Neurotrophic Factor (MANF). MANF expression had been poorly characterized, but Krzeptowski et al. show that it is expressed in clusters of clock neurons in *Drosophila* and that silencing MANF in these neurons alters the rhythm of locomotor activity in flies. They also demonstrate that the captivating phenomenon of circadian-based structural changes in dendritic arbors is disrupted in animals that lack MANF. As a whole, their results indicate that MANF in *Drosophila* is important for both the development of neurons and the maintenance of circadian-related plasticity in neurons.

Finally, all these investigations into neuronal plasticity, survival, and proliferation raise questions surrounding the relationship between brain size and behavior. Coto and Traniello share an opinion piece in which they consider this relationship from an evolutionary point of view, discussing how brain size might evolve in response to the requirements of the environment, social interactions, and cognitive needs. Comparative studies of metabolic and neuroarchitectural scaling can inform our understanding of the relationship between brain scaling, behavioral complexity, and plasticity. They compellingly argue for exploring these relationships in ants. Eusocial insects with complex individual and group cognition, ants vary enormously in size. They propose the use of ants as a model to explore how the traits of metabolism, brains size, and neuroarchitecture correlate with social behavior and scale with body size.

## Author Contributions

HWH wrote the initial draft of this editorial, which was edited by WR and GT. All authors contributed to the article and approved the submitted version.

## Conflict of Interest

The authors declare that the research was conducted in the absence of any commercial or financial relationships that could be construed as a potential conflict of interest.

## Publisher's Note

All claims expressed in this article are solely those of the authors and do not necessarily represent those of their affiliated organizations, or those of the publisher, the editors and the reviewers. Any product that may be evaluated in this article, or claim that may be made by its manufacturer, is not guaranteed or endorsed by the publisher.
